# Standardized approach to result analysis and interpretation of initial experience of coronary computed tomography angiography in a tertiary care hospital

**DOI:** 10.12669/pjms.36.4.2174

**Published:** 2020

**Authors:** Tahira Nishtar, Nadeem Ullah, Tabish Ahmad, Fatima Ahmed

**Affiliations:** 1 Dr. Tahira Nishtar, FCPS Diagnostic Radiology, Associate Professor. Department of Radiology, MTI-Lady Reading Hospital, Peshawar, Pakistan; 2 Dr. Nadeem Ullah, FCPS Diagnostic Radiology. Department of Radiology, MTI-Lady Reading Hospital, Peshawar, Pakistan; 3 Dr. Tabish Ahmad, PGR FCPS Diagnostic Radiology. Department of Radiology, MTI-Lady Reading Hospital, Peshawar, Pakistan; 4 Dr. Fatima Ahmad, Trainee Registrar Diagnostic Radiology. Department of Radiology, MTI-Lady Reading Hospital, Peshawar, Pakistan

**Keywords:** Coronary CTA, Calcium scoring, Bypass graft patency

## Abstract

**Objective:**

To have a systematic standardized approach to performing and interpretation of coronary CT Angiography (CTA) in order to maintain and enhance the diagnostic accuracy of the imaging modality.

**Methods:**

This retrospective observational study was performed in Radiology Department, Lady Reading Hospital, Medical Teaching Institute, Peshawar, Pakistan from August 2018 to September 2019. Patients referred for coronary CT angiography were screened and prepared in radiology department. The examination was performed on Toshiba-160 slice CT Scanner (Prime Aquilion) utilizing standardized protocols tailored towards optimum image acquisition. Interpretation of the images were based on the guidelines provided by the Society of Cardiovascular Computed Tomography (SCCT).

**Results:**

Total 95 CTCA procedures were performed in the department, out of which 85 were included in study showing 49(57%) as normal and 36(42%) were positive for coronary disease. Of the abnormal cases 16(18.8%) had mild disease, nine (10.6%) patients had moderate disease, while severe disease was noted in 11 (12.9%) cases. Coronary quantitative stenosis revealed five cases (5.9%) with LAD involvement only and five (5.9%) with severe triple vessel disease. The remaining varied in degree of stenosis and number of segments involved. Post CABG were 14 cases (16%) and native arteries showed triple vessel disease. CTA for percutaneous stent patency were three (3.5%) cases with 100% stent patency.

**Conclusion:**

Patient selection with tailored protocols are the mainstay for achieving optimal images. This form the basis for accurate interpretation, based on a standardized and systematic approach, utilizing various post processing tools, in order to maintain the high diagnostic accuracy of this semi-invasive, safe imaging modality in a variety of patients suspected of coronary artery disease, coronary artery bypass grafts and stent patency.

## INTRODUCTION

CTCA has become the mainstay imaging modality for assessing the status of coronary vessels. It is preferred over catheter angiography as it is safe, non-invasive imaging modality with short scan time.

The primary mainstay of the coronary CTA procedure is patient selection and appropriate procedural protocols with the aim to acquire ideal images which is a prerequisite for the secondary mainstay, represented by the correct analysis and interpretation of the acquired data using different post processing tools, followed by a formal report with provision of attached selected images to the referring physician. The key is to avoid false positive and false negative findings.

Coronary CTA is today a widely accepted semi invasive imaging modality for both cardiologists and radiologists. The main strength of this imaging modality is dependent on its high negative predictive value,^1^ with patients having a normal study being excluded from undergoing further workup/procedures. However, a false positive result may lead to further workup.

## METHODS

The study is based on the guidelines provided by the Society of Cardiovascular Computed Tomography (SCCT).^2^ Patients referred for CCTA scans to the radiology department, are verified for indication for the procedure. Patient appropriateness for the procedure and selection of tailored protocol is done in the radiology department. Referrals were from wards and outpatient cardiology clinics. All referrals require completion of a safety questionnaire form in radiology department based on the Royal College of Radiologists recommendations. The study was done with the approval of the research ethics committee of Lady Reading Hospital (Ref:No. 355/LRH/MTI dated on December 24, 2019.

Exclusion criteria from CTA study were patients with poorly controlled asthma, atrial fibrillation/flutter. Paediatric patients were also excluded from the study. Other exclusion criteria were poorly controlled tachycardia. All patients were advised to take their routine medications on schedule (including beta-blockers or calcium channel blockers), refrain for eating for four hours prior to the scan and to avoid caffeine containing drinks and other stimulants for six hours prior. Some of the patients were started on oral beta-blockers prior to the scan, which helped in heart rate control.^3^

Patients were checked by trained radiology technicians and vitals recorded. Those patients who had poorly controlled heart rate and other co-morbid conditions were excluded from the coronary CTA study. Minimum 20G peripheral venous cannula was placed in the right arm by radiology technician for the purpose of administering intravenous contrast. On achieving satisfactory heart rate control, patients were positioned on the gantry table and ECG electrodes were placed on the chest wall. Breath-hold techniques were practised prior to image acquisition and prospective ECG gated scans were performed.

Calcium score was obtained for all patients. Scans were performed on a Toshiba 160-slice CT scanner (Prime Aquilion). Iopromide (ultravist 300, 1.5ml/Kg) was used with 0.9% sodium chloride bolus chaser. Initially, a 20mL test bolus of contrast with a 20mL saline push was given, and the remaining contrast was administered timely to maximise contrast dose. The time from contrast injection to image acquisition was 7s.

After post-processing, a Royal college of London, level II certified consultant radiologist using a dedicated Toshiba work station generated a report. “Optimal quality” scans meant all coronary arteries and branches were clearly visualised, calcium score and detailed description of the coronary vessels were given. “Suboptimal/poor quality” meant that the vessels were visualised sub optimally due to artefacts.

Reports for normal scans, with low or normal calcium scores, were issued directly to the referring physician. Positive scans were subject to discussion and detailed reporting. The absolute coronary artery calcium (CAC) score was labelled in accordance to Agaston method, as zero for absent CAC, 1-100 as discrete CAC, moderate CAC as 101- 400 and accentuated as more than 400.^4^ The CAC score is an independent marker of risk for cardiac events.

Luminal stenosis for Obstructive coronary artery disease was defined as a luminal stenosis more than 70% in one or more major epicardial vessel or more than 50% in the left main stem.^5^ Luminal cross-sectional area stenosis were classified as minimal (<25%), mild non-obstructive (25–49%), moderate non-obstructive (50–69%), or obstructive (>70%) disease.^6^

## RESULTS

Ninety five CTCA’s were performed between August 2018 and September 2019. Of these, six (6.3%) paediatrics cases that underwent cardiac CT for congenital heart were excluded from the analysis. Two patients had suboptimal studies due to poor heart rate control; one case was abandoned due to atrial fibrillation and another one due to previous history of contrast allergy. Our study was conducted on 85 patients. Out of the 85 patients, 65 (76%) were male patients and 20 (23%) were female. The coronary artery calcium score and quantitative stenosis was analysed in accordance to coronary artery segmentation model. There were 14 (16%) post CABG cases. The number of grafts, frequency of patency, stenosis and occlusion was analysed. Three patients had percutaneously placed stents, which were assessed for patency.

**Fig.1:**
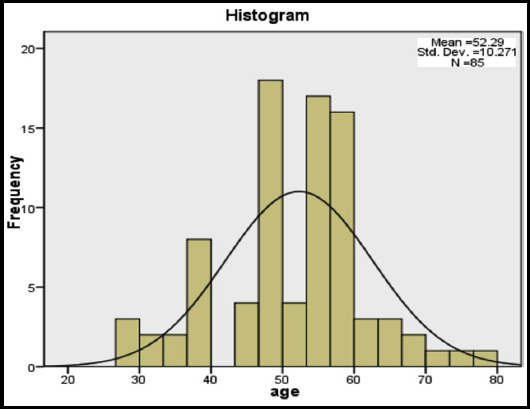
Age distribution.

The most common indication for CTCA was to investigate potential stable CAD accounting for majority of requests. In 42 (49.4%) cases, the CTCA was requested for the assessment of low-risk acute chest pain. In 32 (37.6%) the patients were asymptomatic but the CTCA was performed due to risk factors like hypertension, diabetes mellitus and known coronary artery disease. In the remaining 11 (12.9%), the CTCA was requested for indications like referred epigastric pain mimicking chest pain with palpitations and shortness of breath. The patients ranged in age from 27 to 77 years (Fig.1). A mean age of 52.29 years and standard deviation of ±10 years is calculated using SSPS version 16.

**Fig.2:**
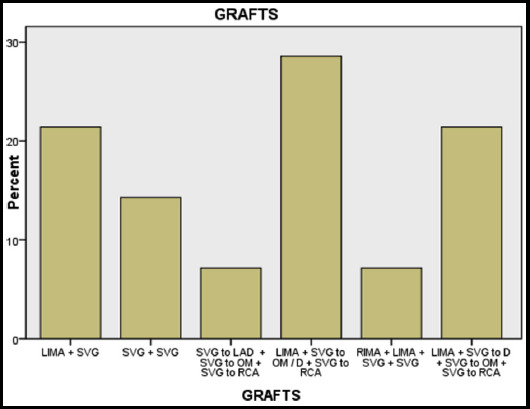
Frequency of various bypass grafts used.

CTCA for Coronary Artery Calcium (CAC) Score based on Agaston method showed49 (57%) individuals as normal and 36 (42%) having positive coronary angiography results (Fig.2). Coronary artery calcium score is illustrated in Table-I. Amongst the positive cases 16 (18.8%) showed mild/discrete CAC score, 9 (10.6%) patients as moderate while severe CAC score was noted in 11 (12.9%) cases.

**Table-I:** Coronary artery calcium score.

Degree of Severity	Calcium scoring	Frequency	Percent	Valid Percent	Cumulative Percent
Normal	Zero	49	57.6	57.6	57.6
Mild	1 to 100	16	18.8	18.8	76.5
Moderate	101 to 400	9	10.6	10.6	87.1
Severe	401 plus	11	12.9	12.9	100.0
	Total	85	100.0	100.0	

Categorization was done as single, two and three vessel disease based on involvement of major arteries like LAD, LCX and RCA. Assessing severity of disease with quantitative analysis of coronary vessels revealed LAD single vessel mild to moderate disease in five cases (5.9%) and severe stenosis in triple vessel disease in five cases (5.9%).

The remaining range of mild, moderate and severe two vessel or mild to moderate triple vessel disease were categorized in accordance to SCCT coronary artery segmentation model and each segment described. The native arteries in post CABG patients were also categorized as triple vessel disease.

Fourteen post CABG patients and three patients with stent placement were assessed for graft and stent patency. All patients with coronary artery stents revealed patent stents with good flow. The post CABG cases were evaluated for the number of grafts, their patency, visibility of stumps / occlusion (Table-III, Fig.2).

**Table-II:** Coronary CT angiography results with degree of stenosis.

Degree of Stenosis of Coronary Artery	Frequency	Percentage	Cumulative Percentage
Mild to Moderate Stenosis LAD	5	5.9%	5.9%
Mild Stenosis LAD and LCx	1	1.2%	7.1%
Mild LAD , severe LCx Stenosis	1	1.2%	8.2%
Severe Stenosis LAD + LCX with Diagonal or OM Branch	1	1.2%	9.4%
Mild LAD + Severe Diagonal / Obtuse Marginal Stenosis	1	1.2%	10.6%
Severe LAD / LCx + Mild RCA Stenosis	1	1.2%	11.8%
Severe LAD + Moderate RCA Stenosis	1	1.2%	12.9%
Moderate LAD + Mild RCA Stenosis	2	2.4%	15.3%
Mild LAD, LCx + Moderate RCA Stenosis	2	2.4%	17.6%
Moderate Stenosis LAD + RCA	1	1.2%	18.8%
Mild stenosis LAD + LCx + RCA	1	1.2%	20.0%
Mild Stenosis LAD + RCA +/- Ramus Intermedius	4	4.7%	24.7%
Severe Stenosis LAD+LCx + RCA, Triple Vessel Disease	5	5.9%	30.6%
Post CABG / Stent placement	17	20.0%	50.6%
Normal study	42	49.4%	100%
Total	85	100. 0%	

(***Abbreviations:*** LAD left anterior descending, LCx left circumflex, D diagonal branch, OM obtuse marginal branch, RCA right coronary artery, Post CABG coronary artery bypass grafting).

**Table-III:** Post CABG bypass graft frequency per patient.

Number of Bypass Grafts	Frequency	Percent	Valid Percent	Cumulative Percent
Double bypass graft	5	5.9	35.7	35.7
Triple bypass graft	4	4.7	28.6	64.3
Quadruple bypass graft	4	4.7	28.6	92.9
5 grafts	1	1.2	7.1	100.0
Total	14	16.5	100.0	
Other patients without CABG including normal study	71	83.5		
Total	85	100.0		

- In our study 5 (35%) patients had two vessel bypass grafts, out of which three patients had both grafts patent, and two patients had one graft patent and one blocked.
- Four patients had triple vessel bypass grafts, out of which two had all grafts patent, and one patient had two grafts patent while one patient had all three grafts blocked.
- Quadruple vessel bypass grafts were seen in four patients, out of which two grafts were patent in two patients, and two patients had only one graft patent.
- It was noted that patients with saphenous vein grafts were likely to be blocked, and the arterial graft LIMA to LAD was more likely to be patent, in these patients.
- One patient had five bypass grafts out of which three were blocked including one arterial graft.
- The various types of bypass graft were also described while reporting CTCA, their patency, stenosis or occlusion as depicted in the table below.

## DISCUSSION

Coronary CT angiography is performed as a semi-invasive imaging modality for assessment of presence or absence of coronary artery disease.^7^ Invasive coronary angiography is still the gold standard investigation for both imaging of emergency and elective coronary artery intervention.^8^ However, because of the risk of complications, coronary CTA is considered an imaging modality of choice having a high diagnostic accuracy in patients suspected of coronary artery disease.^9^ Its diagnostic accuracy is comparable with invasive coronary angiography forming the basis for coronary CTA as the initial investigation by the National Institute for Health and Care Excellence (NICE) for stable coronary artery disease and atypical angina in the UK.^10^

The key to acquire an optimum cardiac CT image is based on advanced CT protocols and techniques. This is dependent on fast acquisition requiring, precise scan timing ensuring optimum contrast opacification which is about 250-300 HU in order to delineate low density soft atherosclerotic plaques having density of 40 HU from intermediate fibrous plaques (90 HU) and calcified plaques (>130 HU).

Imaging and the field of view (FOV) is set accordingly. Successful imaging is dependent on faster scanning and slower heart rate. In other words, heart rate control is critical for the examination which is acquired by beta blockers to have an ideal heart rate of 50-59 beats/minute. IV metoprolol is safe with increments given every 5 minutes to a total dose of 15-25mg with ECG gating. Multiple phases are acquired over a cardiac cycle for optimum images of coronary arteries, hence image data is acquired throughout the cardiac cycle. Standard mAs are 500-550 mAs with 120kV with slice thickness of as low as 0.5mm.^7^ One of the main advantages of coronary CTA over catheter angiography is acquisition of multi planar reconstructed images, the quality of which is inversely proportional to the slice thickness, hence an ideal CTA has an increased negative predictive value.^9^ Around the year 2000, multi detector CT scans were introduced making coronary imaging free of motion artefacts. Standard radiation dose is 1.5-5mSv,^3^ dependent on the patient habitus and CT protocols as well as the type of CT system used but with tailored protocols dose can be reduced to below 1.0mSv.

Suboptimal non diagnostic indeterminate scan is acquired in overweight patients, those who cannot hold breath and with fast heart rate. Cardiac CT assessment encompasses a range of parameter’s as shown in Table-IV:

**Table-IV:** Cardiac CT review methodology.

Series	Check for
	• Calcium score
	• Any other calcification
	• Pericardium, hiatus hernia, etc.
	• Pulmonary/pleural/hiatus abnormalities
***Coronary artery analysis***	
Step 1: Coronal Aorta	• Image quality: contrast, noise, step artefacts
	• Aorta and aortic root: size and any plague
Step 2: LVOT	• Aortic root: measure if dilated
	• Left atrium size
Step 3: AV double oblique	• AV morphology
	• Coronary origins
	• LA appendage for any thrombus
Step 4: Long & short axis views	• Chamber size
	• Myocardial thickness if ED phase
	• Myocardial enhancement
	• Atrial and ventricular septum
	• LAD course
Step 5: Vessel walking	• Go through LMS, LAD, Diagonals, CXA, Obtuse Marginals, RCA, and PDA for general morphology, branching pattern and plaque distribution
Step 6: Vessel Analysis	• Use curved MPR on each vessel to analyse
	• Any plaques
	• Characterize the plaques
	• Evaluate the degree of stenosis

We analysed the number and types of bypass grafts, their patency and the presence of stumps or stenosis, and status of native vessels. There was occlusion in 41% of bypass grafts, although the time of surgery varied among patients from one to 15 years. Although there are many causes of venous bypass graft dysfunction. The two major causes of graft failure within the first year after surgery are intimal hyperplasia and thrombosis, with an occlusion rate of 10–15%. And after the first year, atherosclerosis mechanisms predominate. After five years, atherothrombotic occlusion of venous grafts accounts for a reduced patency rate. It has traditionally been estimated to range between 40% and 60% at 10-12 years.^11^

Study show that evaluation of coronary bypass grafts by CTA is highly accurate in predicting the findings seen on ICA with a meta-analysis, of 16- and 64-slice CTAs quoting a sensitivity of 98% and specificity of 97% for graft stenosis or occlusion.^12^

CTA increases the detection of graftable LAD and thus favours CABG in patients which, otherwise, seem poor candidates on conventional angiography thereby increasing the survival of patients with coronary heart disease.^13^

Invasive angiography is more sensitive than CT angiography but both have equal specificity for the detection of stenotic lesions in the distal segments of four main coronary arteries.^14^

### Recommendations

Appropriate referrals, patient preparation and scan quality remain significant factors in running a CTCA service. An MDT approach would be a key to the delivery of a cardiac CT services. In addition, use of intravenous beta blockers needs to be encouraged for optimum image quality in a controlled, safe environment. It is also recommended to get calcium score and coronary CT angiography done in the same setting as the absence of calcium or low calcium does not exclude significant coronary stenosis.^15^

## CONCLUSION

Patient selection with tailored protocols are the mainstay for achieving ideal images required for accurate interpretation based on a standardized and systemic approach, utilizing various post processing tools, in order to maintain the high diagnostic accuracy of this semi-invasive, safe imaging modality in patients suspected of coronary artery disease, coronary artery stent and graft patency.

### Authors’ contribution

**TN:** Conceived, data collection, manuscript writing & editing, review and final approval and integrity of the work.

**NU:** Manuscript writing, editing of manuscript, references and accuracy.

**TA:** Manuscript writing and editing of references.

**FA:** Data analysis, manuscript writing, editing of references and accuracy.
